# Ipsilateral Distal Both Bone Forearm and Lateral Humeral Condyle Fractures With Posterolateral Elbow Dislocation: A Rare Injury in a Child

**DOI:** 10.7759/cureus.74002

**Published:** 2024-11-19

**Authors:** Mustafa Özyildiran

**Affiliations:** 1 Department of Orthopedics and Traumatology, Sandıklı State Hospital, Afyonkarahisar, TUR

**Keywords:** elbow dislocation, forearm fracture, fracture with elbow dislocation, lateral humeral condyle fracture, pediatric elbow dislocation

## Abstract

Lateral humeral condyle fractures are common in children, but concomitant elbow dislocation is rare. This case report involves a 10-year-old girl with an ipsilateral distal both-bone forearm fracture and a lateral humeral condyle fracture accompanied by a posterolateral elbow dislocation. Closed reduction of the elbow was performed in the emergency department without delay, and the patient was operated on as soon as possible. Closed reduction percutaneous pinning (CRPP) was performed for the distal radius fracture. The Weiss type III lateral humeral condyle fracture was reduced through a lateral approach, and fixation was performed using divergent pinning with two Kirschner wires. K-wires were removed, and the motion was initiated postoperatively in the fourth week. The patient obtained favorable postoperative results and regained full range of motion in both the elbow and wrist. Any complications, such as nonunion, malunion, cubitus varus, or avascular necrosis, were not observed.

## Introduction

Lateral humeral condyle fractures are the second most common fractures at the elbow in the pediatric population, following supracondylar humerus fractures, accounting for 12% to 17% of all distal humerus fractures [1,2]. Elbow dislocation is a common injury in children, and accompanying medial epicondyle fractures are well documented in the literature [3,4]. However, dislocation of the elbow associated with lateral condyle fracture is very rare, and the literature is mostly limited to case reports and small case series [1,5-7]. Pediatric lateral humeral condyle fractures are intra-articular injuries that carry considerable risks of complications, including malunion, delayed healing, nonunion, stiffness, and avascular necrosis [5,8].

Combined distal humerus and ipsilateral forearm bone fractures are uncommon injuries in children. Cases of supracondylar humerus fractures accompanied by ipsilateral forearm fractures have been reported in the literature [9,10]. This is a severe injury, with some researchers documenting a high incidence of compartment syndrome in such cases [10,11]. Here, we present a case of a child with distal forearm and lateral humeral condyle fractures associated with a posterolateral elbow dislocation. To date, no cases of this type of combined fracture and associated posterolateral elbow dislocation have been reported in the literature. This case report describes the surgical treatment of this rare injury, reports the clinical outcomes, and provides a review of the existing literature.

## Case presentation

**Figure 1:**
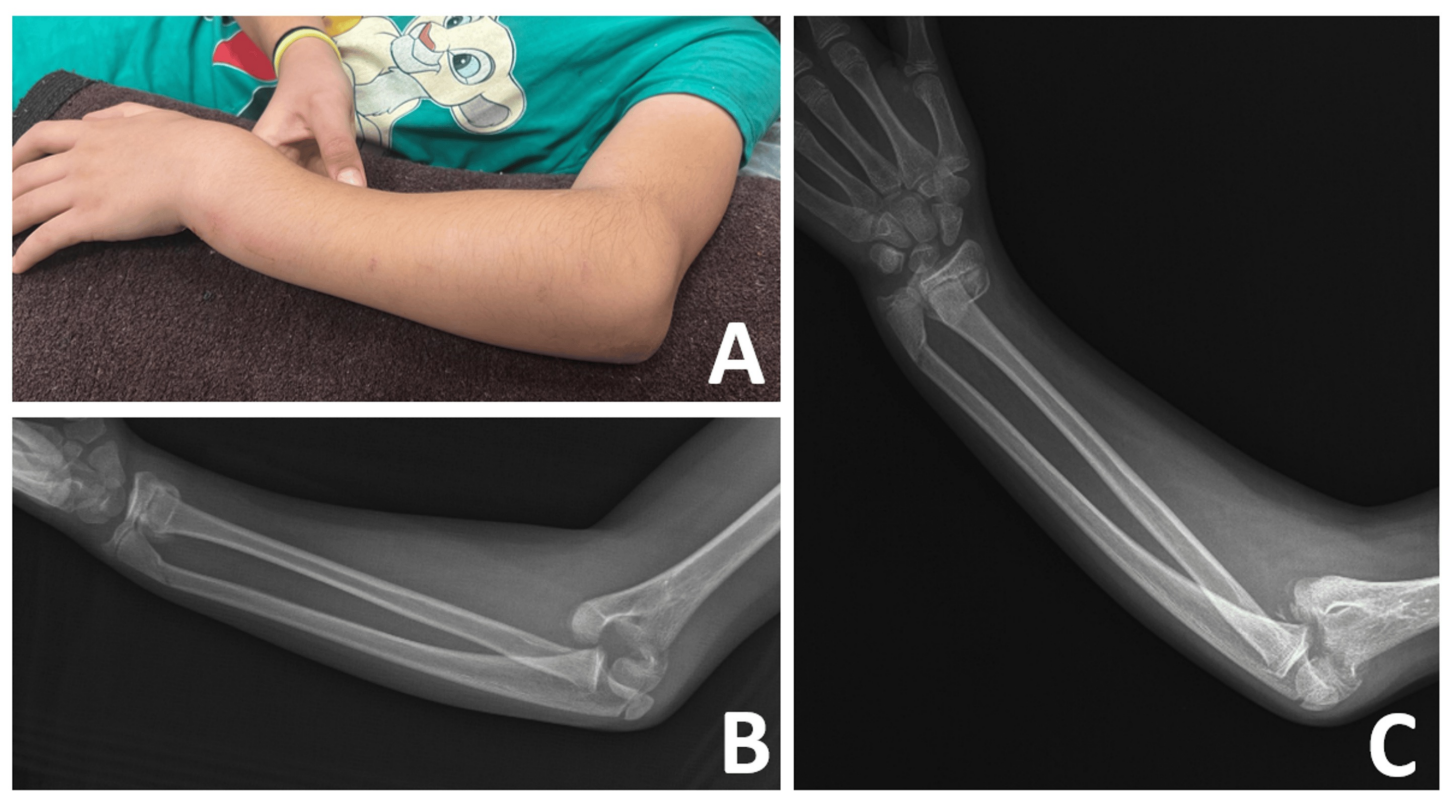
Initial photograph and radiographs of the left upper extremity in the emergency department (A) The photograph taken by the emergency physician shows deformities in the left elbow and wrist. (B and C) Initial radiographs demonstrate distal both-bone forearm and lateral humeral condyle fractures with posterolateral elbow dislocation.

A 10-year-old girl fell from a wall with a height of two meters onto her left outstretched hand. It was learned that the elbow was in extension, and the forearm was in the supination position during the fall. The physical examination revealed swelling and deformities in the left wrist and elbow, accompanied by severe pain (Figure 1A). The neurovascular status of the affected limb was intact, and there was no injury to any other part of the body. Initial radiographs demonstrated a distal both-bone forearm fracture and a lateral humeral condyle fracture, along with posterolateral elbow dislocation (Figures 1B, 1C).

**Figure 2:**
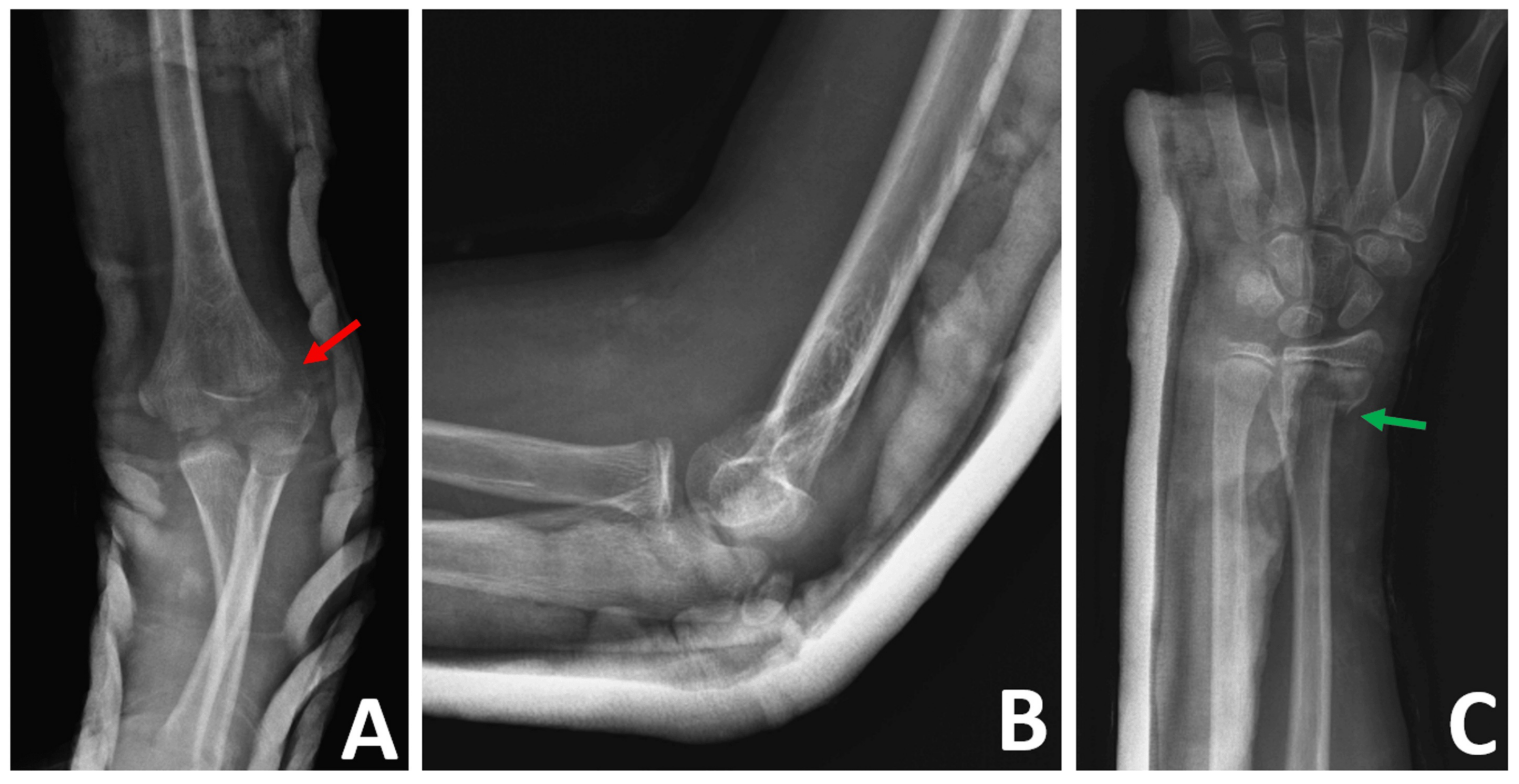
Preoperative radiographs of the left elbow and wrist after closed reduction and splinting (A) Anteroposterior radiograph of the left elbow shows a Milch type II fracture of the lateral condyle, with displacement greater than 4 mm. Red arrow indicates the fracture line. (B) The lateral radiograph demonstrates that the elbow is in a reduced position after a closed reduction maneuver. (C) Anteroposterior radiograph of the left wrist shows a distal radius metaphyseal fracture (green arrow).

Following the initial examination and X-ray assessment conducted by the emergency physician, the patient was consulted by the orthopedic specialists. Closed reduction of the elbow under conscious sedation was performed urgently in the emergency department, followed by long-arm splint immobilization. The radiographs taken after the reduction were examined. The fracture line in the lateral humeral condyle extended medially into the trochlear groove, with displacement greater than 4 mm. This fracture was classified as Milch type II and Weiss type III (Figure 2). The patient was admitted to the orthopedic service for surgical management.

**Figure 3:**
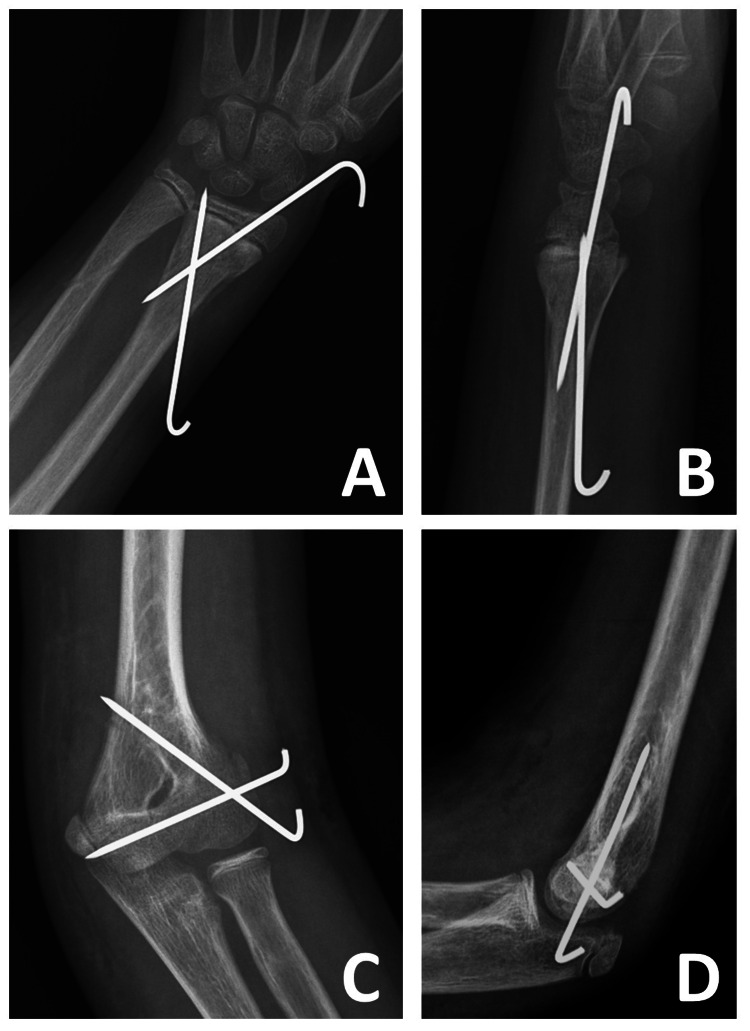
Radiographs taken at four weeks postoperatively (A, B) Anteroposterior and lateral radiographs of the left wrist. (C) Internal oblique radiograph of the left elbow. (D) Lateral radiograph of the left elbow.

The surgical procedure was performed by a surgeon experienced in pediatric orthopedics as soon as starvation status allowed access, approximately six hours after the initial presentation. A single intravenous dose of prophylactic antibiotics (1000 mg cefazolin) was administered. General anesthesia was administered, and the procedure was performed under a tourniquet. The forearm fracture was addressed first. Closed reduction of the distal radius fracture was performed using indirect reduction techniques. A Kirschner wire was advanced through the fracture line and used as a joystick to manipulate the displaced fragment. After closed reduction, the distal radius was fixed with two 1.8 mm Kirschner wires. The ulna was not fixed, as stable anatomical reduction had been achieved by pinning the radius. The fracture of the lateral humeral condyle was identified and reduced through a lateral approach, utilizing the interval between the brachioradialis and triceps. During the dissection and reduction, the posterolateral soft tissue attachments of the lateral condyle were preserved to reduce the risk of avascular necrosis of the capitellum. The reduction was stabilized with two divergent 1.8 mm Kirschner wires, one parallel to the joint line and the other at maximum perpendicularity to the fracture line. It was preferred to leave the Kirschner wires exposed rather than burying them subcutaneously. Following the fixation of the lateral condyle, the elbow was stable throughout its full range of flexion and extension. The ends of the K-wires were cut and bent (Figure 3). Subsequently, a long arm splint in a neutral position was applied. At four weeks postoperatively, the long arm splint and K-wires were removed in the outpatient department without anesthesia. Following the removal of the pins and splint, joint movement exercises were started immediately.

**Figure 4:**
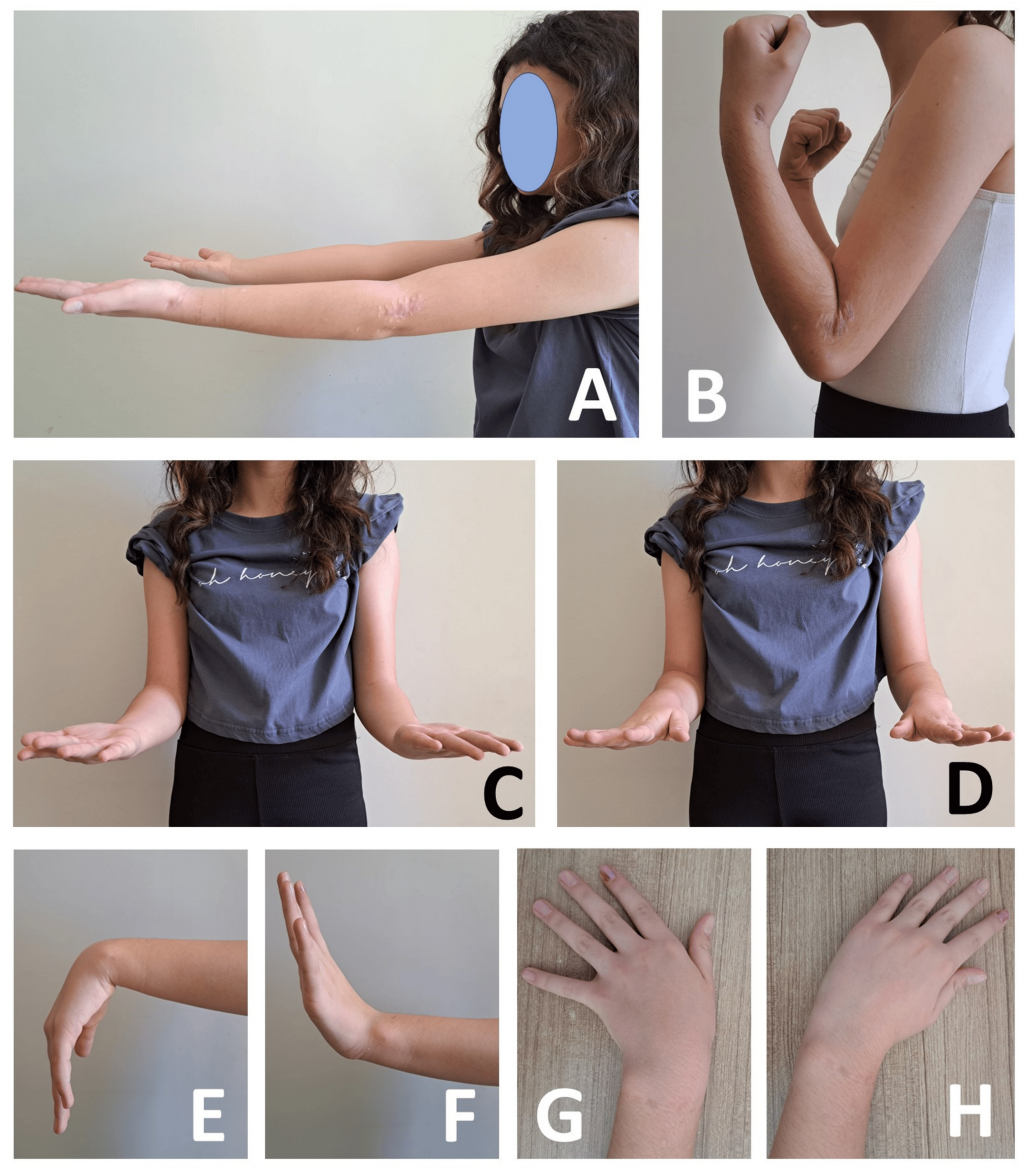
Postoperative range of motion in the left elbow and wrist The patient demonstrated a full range of motion in the left elbow and wrist at six months postoperatively. (A) Extension of the elbow. (B) Flexion of the elbow. (C) Supination of the forearm. (D) Pronation of the forearm. (E) Flexion of the wrist. (F) Extension of the wrist. (G) Ulnar deviation. (H) Radial deviation.

Physical therapy and rehabilitation support were initiated three months after surgery. The patient was observed to have achieved a full range of motion in the elbow and wrist at the six-month follow-up (Figure 4). Clinical scores were excellent, with a Disabilities of the Arm, Shoulder, and Hand (DASH) score of 5.8 (range: 0-100). 

**Figure 5:**
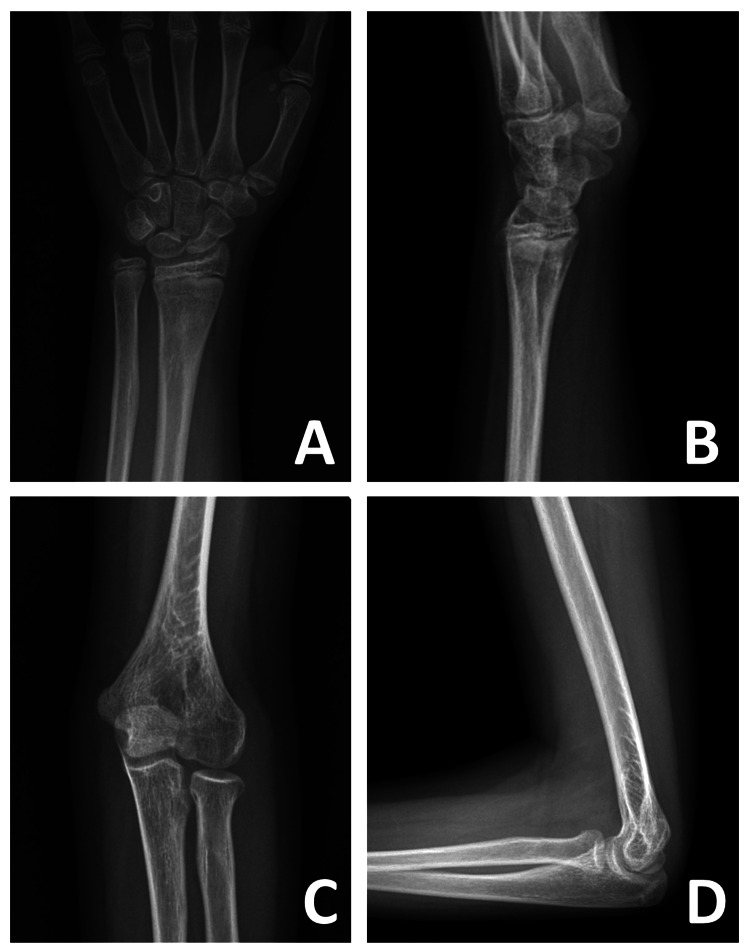
Radiographs taken at one-year postoperative follow-up (A,B) Anteroposterior and lateral radiographs of the left wrist. (C,D) Anteroposterior and lateral radiographs of the left elbow.

The final follow-up at 12 months showed good functional and radiological outcomes. Any complications, such as nonunion, malunion, cubitus varus, avascular necrosis, or fishtail deformity, were not observed (Figure 5). The patient returned to her normal daily activities without any problems.

## Discussion

Lateral humeral condyle fractures are the second most common pediatric elbow fracture after the supracondylar humerus fracture [1,2]. However, the association of a lateral condyle fracture with an elbow dislocation is very rare in children. Pediatric elbow dislocations are often associated with a medial epicondyle fracture [5]. In cases of lateral condyle fractures accompanied by elbow dislocation, it is known that the direction of dislocation is usually posteromedial [1,3,12]. In a study of 789 lateral condyle fractures in children, Silva et al. reported 12 cases (1.5%) with an associated dislocation, of which only one was posterolateral while the other 11 cases were posteromedial [12]. Masquijo et al. reported that in their multicenter cohort study of 23 cases with concomitant lateral humeral condyle fractures and elbow dislocations, only 5 cases had a posterolateral dislocation [1]. As stated in the literature, the association of a lateral condyle fracture with a posterolateral elbow dislocation is exceedingly rare in children. In addition to these, our case also had an accompanying distal both-bone forearm fracture. To our knowledge, this is the first case report of the ipsilateral distal both-bone forearm and lateral humeral condyle fractures accompanied by posterolateral elbow dislocation. The injury occurred due to a fall onto an outstretched hand, combined with a valgus force (push-off mechanism) applied to the forearm while the elbow was extended. An older patient who has reached skeletal maturity and experiences the same mechanism of injury may have instead suffered from posterolateral rotatory instability and a terrible triad fracture.

Lateral humeral condyle fractures are associated with a greater risk of complications than other types of elbow fractures, highlighting the importance of accurate diagnosis and appropriate management [3,5,8]. Milch was the first to characterize the patterns of lateral condyle fractures [13]. According to the Milch classification, Type I fractures have a fracture line that is lateral to the trochlear groove, whereas Type II fractures extend medial to the trochlear groove. The Milch type I lateral condyle fracture is considered more stable because the intact capitellotrochlear groove acts as a lateral buttress for the coronoid-olecranon ridge of the ulna [3,13]. Weiss et al. described another classification system based on the degree of displacement and the integrity of the articular surface. According to the Weiss classification, Type I fractures show less than 2 mm of displacement, Type II fractures have 2 mm or more displacement with intact articular cartilage, and Type III fractures involve 2 mm or more displacement with disrupted articular surface integrity [14]. The Weiss classification determines whether surgical intervention is required. Type I, undisplaced stable fractures are treated non-operatively with cast immobilization. Type II fractures, which have an intact articular hinge, may be treated with closed reduction and percutaneous pinning. Type III fractures, which are unstable, malrotated, and displaced by more than 2 mm, are usually managed with open reduction [2,14]. 

There are several case series in the literature indicating that closed reduction and pinning can be performed even in Weiss Type III fractures [2,15]. However, open reduction is widely recommended in the literature for all Type III displaced lateral condyle fractures. It is known that this method has better clinical outcomes compared to closed reduction [1,2,5]. In our case, the fracture pattern was classified as Milch Type II and Weiss Type III. Open reduction was performed in accordance with the literature. The lateral approach is recommended in the surgical techniques, emphasizing that dissection of the fracture should be performed anterior to the joint. Avoidance of soft tissue dissection of the posterior aspect of the fragment is highlighted in the literature to prevent avascular necrosis [2,3,16]. Fixation methods after open reduction include Kirschner wires or screws, with no consensus on the optimal technique [1,5,16]. Schlitz et al. reported that screw fixation was stronger than divergent pinning in a lateral condyle fracture on a synthetic bone model [17]. Masquijo et al. reported that there were no differences in the complete arc of motion between patients treated with K-wires and cannulated screws [1]. Wendling-Keim et al. stated that K-wire fixation of pediatric lateral condyle fractures resulted in a lower complication rate than screw fixation [18]. Leonidou et al. advocated for open reduction and K-wire fixation in all displaced lateral condyle fractures, as their results demonstrated successful union and good functional outcomes in all 67 patients, with no significant complications [2]. Bloom et al. conducted a biomechanical study on lateral humeral condyle fracture pinning. They found that optimally divergent Kirschner wires (spread at 60°) provided significantly greater stability than less divergent pins in torsional loading among 2-pin configurations [19]. In line with the literature, we applied divergent pinning with two K-wires.

Combined distal humerus fracture with ipsilateral forearm bone injury is considered a high-energy fracture. The force is so great that a single fracture cannot absorb all the energy of trauma, increasing the risk of soft tissue injury [9,11]. Case series of supracondylar humerus fractures and concomitant ipsilateral forearm fractures have been reported in the literature [9,10]. Some of these studies documented a high incidence of compartment syndrome in such cases [10,11]. Blakemore et al. conducted a retrospective study and examined 43 children with ipsilateral fractures of the humerus and forearm. Of these 43 children, three (7%) developed compartment syndrome and required forearm fasciotomies [11]. In our case, there was no supracondylar fracture. However, there was a lateral condyle fracture and elbow dislocation accompanying the forearm fracture. Considering the injury pattern and high-energy trauma, there was a risk of compartment syndrome in our case. Closed reduction of the elbow was performed in the emergency department without delay. There was no excessive swelling or other signs of compartment syndrome after the closed reduction procedure. The patient was operated on as soon as the starvation status allowed access. The distal radius was reduced and fixed first because we believe that leaving the forearm dangling during the reduction of the distal humerus could cause more soft tissue injury to the forearm and increase the risk of compartment syndrome. Postoperatively, swelling in the forearm and wrist persisted for 48 hours; however, there were no other signs of compartment syndrome, such as persistent pain, paresthesia, or diminished pulses. The patient was closely monitored, and the swelling subsided without the need for a fasciotomy. Recognition of this type of injury and prompt closed reduction of the elbow preoperatively is important to decrease the risk of compartment syndrome.

Elbow stiffness is known to be one of the most common complications after a distal humerus fracture [1,3,5,12]. The presence of an intra-articular fracture and soft tissue injuries would increase bleeding and the release of inflammatory agents that play a role in the repair process. In some cases, excessive scar formation and joint capsule contracture may occur in response to severe trauma. Prolonged immobilization may predispose the joint to an increase in collagen cross-linking and subsequent joint capsule contracture [1,20]. Initiating physiotherapy as soon as possible after the immobilization period is crucial, as it has been demonstrated to be associated with fewer residual symptoms and quicker improvements in range of motion and strength [2]. In our case, following the removal of the pins in the fourth week postoperatively, joint movement exercises were started immediately. At three months postoperatively, there were 20 degrees of loss of extension in the elbow. However, following physical therapy, the patient achieved a full range of motion in the elbow and wrist at the six-month follow-up.

## Conclusions

A distal both-bone forearm fracture and a concomitant lateral humeral condyle fracture associated with a posterolateral elbow dislocation a very rare injury in the pediatric population. This case study presents the successful management of this rare injury through prompt reduction of the elbow dislocation and timely anatomic reduction and fixation of the distal humerus articular surface. Based on our own experience and a literature review, we recommend open reduction and K-wire fixation for the Weiss type III displaced lateral condyle fracture and closed reduction and percutaneous pinning (CRPP) for the distal radius fracture. Anatomic reduction of the elbow joint and early motion exercises are important for achieving satisfactory clinical outcomes. Delayed recovery of elbow motion may be observed; however, a full range of motion can be achieved in the long term with effective physiotherapy.
